# Small Bowel Obstruction Secondary to Endometriosis Affecting the Appendix in a Patient With a Virgin Abdomen

**DOI:** 10.7759/cureus.79717

**Published:** 2025-02-26

**Authors:** Nirupma Mehrotra, Angelina Trovato, Christian Massier

**Affiliations:** 1 General Surgery, Cleveland Clinic South Pointe Hospital, Warrensville Heights, USA

**Keywords:** appendectomy, endometriosis, internal hernia, small bowel adenocarcinoma, small bowel obstruction

## Abstract

A 62-year-old otherwise healthy Caucasian female patient presented to the emergency department with a one-day history of severe abdominal pain, accompanied by nausea and non-bloody emesis. She did not report any prior similar episodes and denied any history of abdominal surgery. Although she did not report a history of diagnosed endometriosis, she did report a history of dysmenorrhea. Laboratory tests in the emergency room showed mild leukocytosis. A computed tomography (CT) scan of the abdomen showed a small bowel obstruction with a transition point in the right lower quadrant with associated reactive enteritis. A diagnostic laparoscopy was subsequently performed. Intraoperatively, we found an adhesive band from the appendix to the sigmoid colon resulting in an internal hernia. Laparoscopic adhesiolysis was performed to release the adhesive band, and since the appendix looked a little abnormal, the decision was made to perform an appendectomy. Her surgical pathology showed endometriosis near the appendiceal tip, estrogen receptor-positive (ER+). Her peritoneal fluid cytology was negative for malignant cells. The patient did well post-operatively and had no complications.

## Introduction

Background

Endometriosis is a condition where endometrial glands and stroma, which create the lining of the uterus, grow outside the uterine cavity [1]. These lesions are commonly found in the pelvis but can be found anywhere including the bowel, diaphragm, and pleural cavity [2]. Endometriosis is a fairly common condition and is generally non-malignant. Ectopic endometrial tissue and resultant inflammation can cause dysmenorrhea, dyspareunia, chronic pain, and infertility [2]. The first-line treatment for endometriosis is medication management; however, surgery is preferred in refractory cases [1].

Bowel obstruction results from the obstruction of normal flow of intraluminal contents, leading to bowel dilation proximal to the blockage, and decompressed bowel distal to the blockage. Compromised blood flow to the intestinal tissue due to excessive bowel dilation or strangulation of the mesentery can result in increased ischemia, necrosis, and perforation of that area of the bowel [3]. Additionally, it can lead to significantly increased mortality. Small bowel obstruction in particular is fairly common and can result from either mechanical or functional obstruction. Intraperitoneal adhesions, tumors, and complicated hernias account for 90% of mechanical small bowel obstructions [3]. Once a patient is found to have small bowel obstruction, it is essential to determine the etiology of the obstruction to prevent further recurrence or complications. Small bowel obstruction due to endometriosis, although possible, is exceedingly rare.

In this article, we are going to present a case of a patient who presented to the emergency room with a chief complaint of severe abdominal pain, nausea, and vomiting. During her hospital admission, the patient was determined to have a small bowel obstruction due to an unknown etiology. She was taken to the operating room for a diagnostic laparoscopy to explore the cause of her small bowel obstruction. Intraoperatively, the patient was found to have an internal hernia suspected to be causing her small bowel obstruction. As a result, she underwent laparoscopic adhesiolysis and appendectomy. The pathology showed focal endometriosis on the appendix.

## Case presentation

A previously healthy 62-year-old Caucasian female patient, with a past medical history significant for monoclonal gammopathy of undetermined significance (MGUS) and dysmenorrhea, presented to the emergency room with a one-day history of severe abdominal pain, accompanied by nausea and non-bloody emesis. Her pain was present in the upper abdomen, radiating down to the midline and lower abdomen. She tried Gaviscon and Tums with no relief. Apart from these complaints, she also endorsed having chills, lots of belching the few days prior to her presentation, orthopnea, loss of appetite, and a sour taste in her mouth. These symptoms were initially intermittent but became constant at the time of her presentation. She denied having any bowel movements or passing flatus for the preceding few days but reported that she had been having regular bowel movements prior to this episode. Additionally, she reported feeling unwell and “coming down with a cold and sore throat” the week prior to this. She denied any diarrhea, dysphagia, or melena. She did not have a history of chronic aspirin or ibuprofen use. She did not have a history of abdominal surgery. Her last colonoscopy was in 2022, which showed one ascending colon polyp, which was subsequently removed. At the time of presentation, her vitals were stable. On examination, her abdomen was soft, she had tenderness to palpation to the left of her umbilicus, and bowel sounds were present in all four quadrants.

Investigation

On labs, she was found to have mild leukocytosis, no acute anemia, and mild hypokalemia. Her lipase was within normal limits. Her urinalysis was not concerning for a urinary tract infection. A carcinoembryonic antigen assay (CEA) was ordered to rule out small bowel adenocarcinoma (normal at 1.1). Additionally, a lactate dehydrogenase (LDH) test was ordered to rule out small bowel lymphoma (slightly elevated at 226, prior value of 251 two years ago, and a normal value between that time frame). This was being monitored by her hematologist/oncologist following her for her diagnosis of MGUS. Lastly, a urine 5-hydroxyindoleacetic acid (5-HIAA) was ordered to rule out a carcinoid tumor, the result for which did not return until after she was discharged (normal at 5).

**Figure 1:**
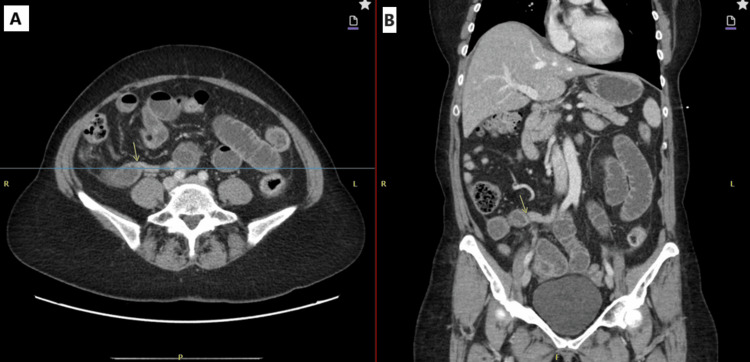
CT abdomen/pelvis: (A) axial and (B) coronal views CT: computed tomography

For imaging, a computed tomography (CT) scan of the abdomen and pelvis performed on the day of admission (Figure 1) showed a small bowel obstruction with a transition point in the right lower quadrant with associated reactive enteritis.

**Figure 2:**
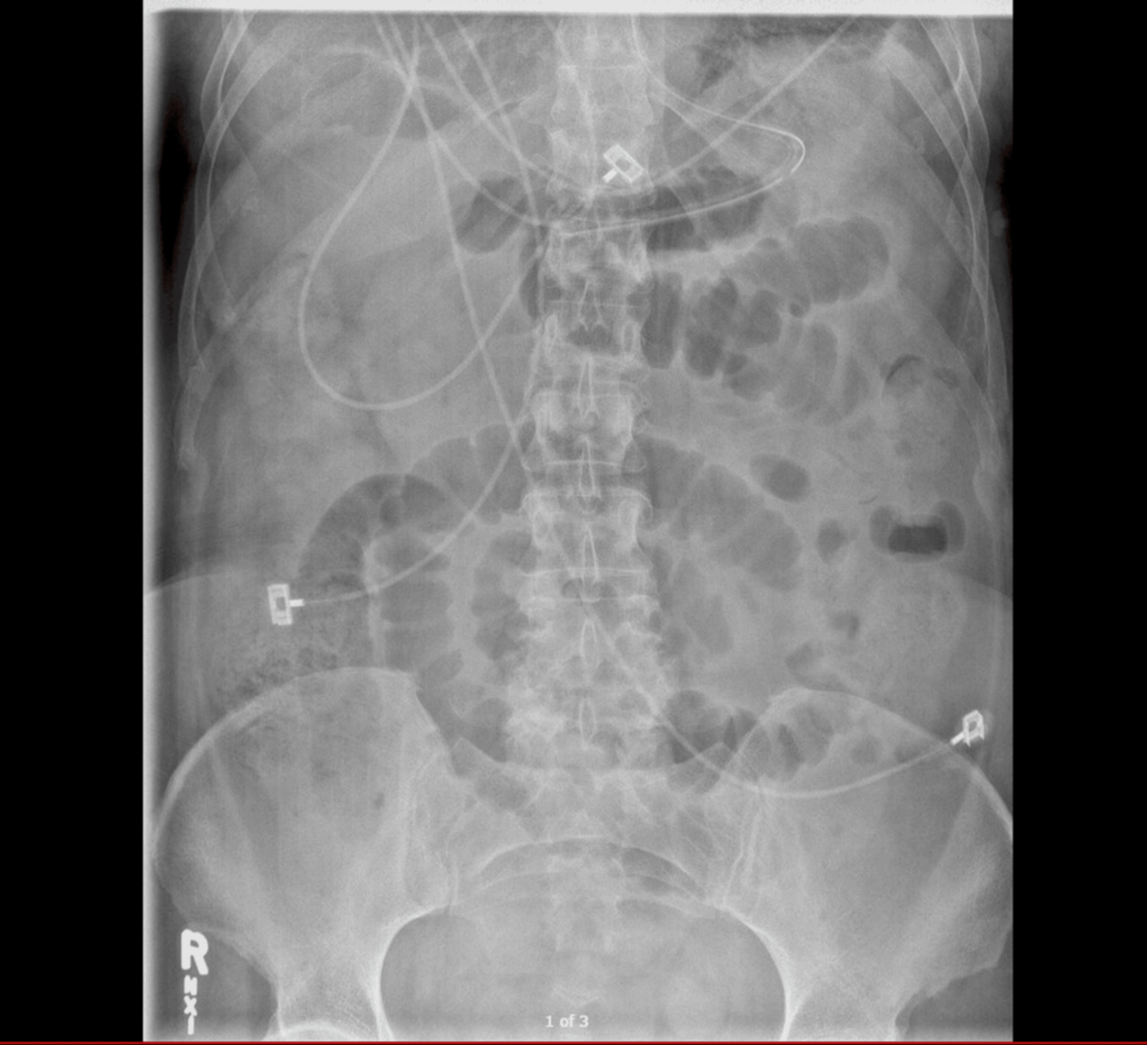
Upright KUB X-ray hospital admission day two KUB: kidney, ureter, and bladder

An upright kidney, ureter, and bladder (KUB) X-ray taken on the second day of admission (Figure 2) was significant for widespread small bowel distention, ileus vs. obstruction. 

**Figure 3:**
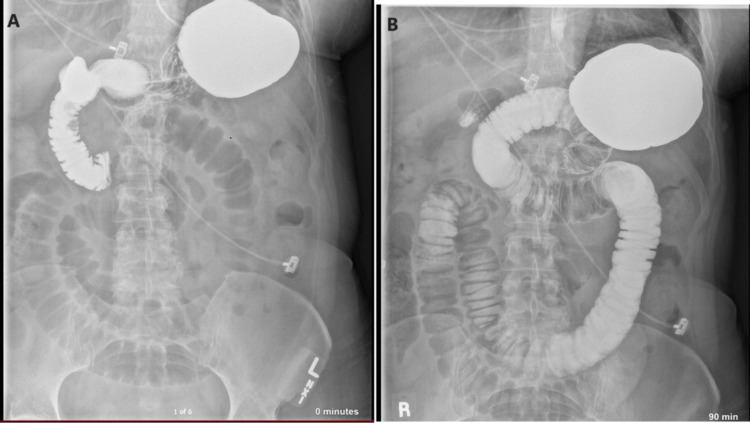
Small bowel series: (A) 0 minutes and (B) 90 minutes

**Figure 4:**
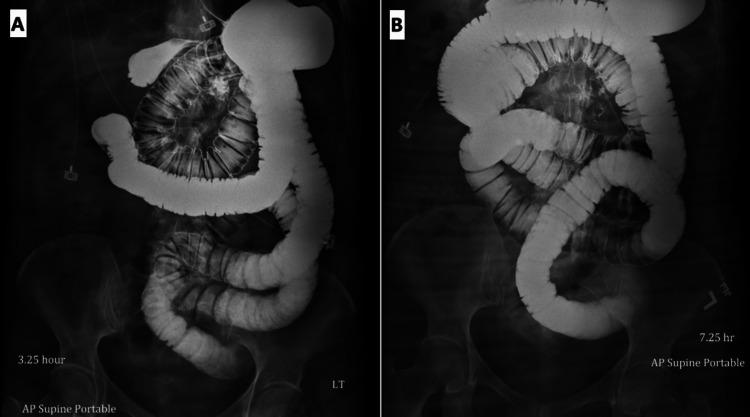
Small bowel series: (A) 3.25 hours and (B) 7.25 hours

A small bowel follow-through was done on the same day, which showed high-grade small bowel obstruction. The X-ray images from 0 minutes, 90 minutes, 3.25 hours, and 7.25 hours are shown below (Figures 3, 4).

**Figure 5:**
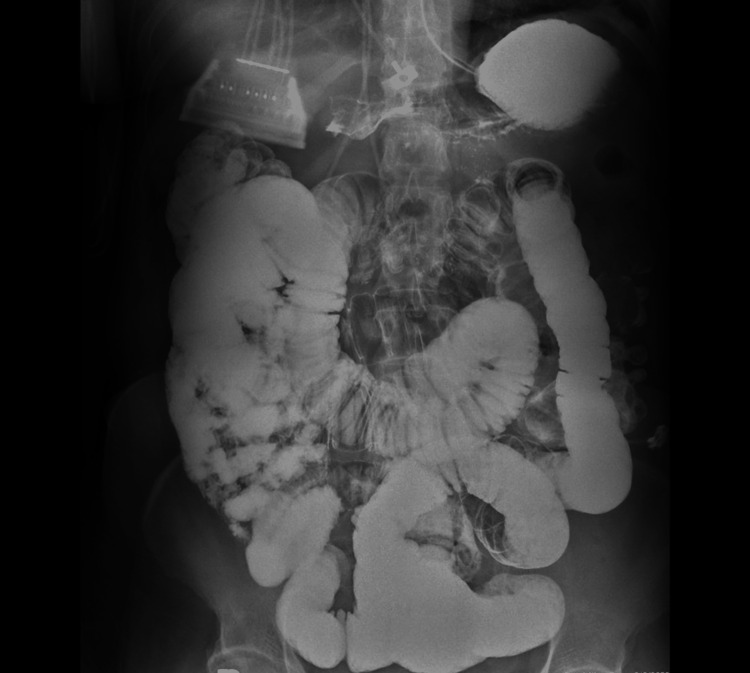
Upright KUB X-ray hospital day three KUB: kidney, ureter, and bladder

A follow-up KUB X-ray on hospital day three (Figure 5) showed some contrast in the proximal colon, but most was seen in the small bowel with unchanged dilation.

Differential diagnosis

Given her clinical history of no surgeries, hernias, etc., and that imaging showed a high-grade small bowel obstruction, we had concerns for possible malignancy such as a neuroendocrine tumor, lymphoma, and small bowel adenocarcinoma. Given her lack of history of abdominal surgeries and concurrent hernias, the most common causes of small bowel obstruction were lower on the differential diagnosis.

Treatment

**Figure 6:**
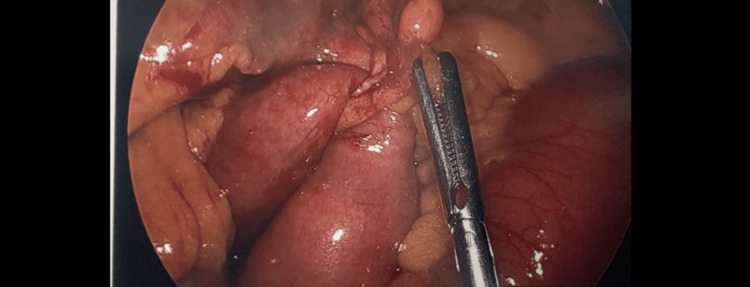
Intraoperative abdominal/pelvic cavity

Initially, non-operative management was employed on hospital admission day one. A nasogastric (NG) tube was placed in order to decompress the bowel. The NG tube output showed brown content. About a few hundred cc output was reported in the emergency room on hospital day one. On hospital day two, she only had about 75 cc NG tube output. On hospital day three, there was again minimal NG tube output (25 cc), minimal resolution of her symptoms, and no return to bowel function. It was collectively decided with the patient that she would benefit from a diagnostic laparoscopy. During laparoscopy, she was found to have proximal dilated bowel with serosal petechia in the distal ileum. About 50 cm proximal to the cecum, we found a band causing an internal hernia. This band was formed by the appendix and adhered to the sigmoid colon. This is illustrated in the image taken intraoperatively (Figure 6).

These adhesions were bluntly taken down, thereby releasing the internal hernia. Her inflamed appendix was concurrently resected. The bowel was run proximally with no notable hemorrhage or ischemia. The resected appendix sample was sent to pathology. She also had a significant amount of peritoneal fluid, which was suctioned out, and a sample was taken to send for cytology.

Outcome and follow-up

Following the diagnostic laparoscopy, the patient did well post-operatively. She had a bowel movement the following day and was discharged post-operative day two. She presented to the clinic for a follow-up two weeks after her surgery. At that time, she complained of no symptoms. Her abdomen was soft, non-tender, and non-distended. Her laparoscopic incision sites were healing well without any evidence of infection. Her surgical pathology showed endometriosis near the appendiceal tip, estrogen receptor-positive (ER+). Her peritoneal fluid cytology was negative for malignant cells.

## Discussion

Endometriosis most commonly affects pelvic sites and organs surrounding the uterus and less often affects extra-pelvic organs such as the intestines and the appendix. Endometriosis affecting the appendix causing a small bowel obstruction is extremely rare. It is estimated that less than 1% of intestinal endometriosis leads to an obstruction, most commonly in the rectosigmoid colon [4]. Although patients with endometriosis affecting the gastrointestinal tract are generally asymptomatic, some may experience local inflammation leading to fibrosis and the formation of adhesions, as in the case of this patient.

The etiology of endometriosis is still widely debated. There are several theories out there that explain the origin of endometriosis, the most common of them being retrograde menstruation. The theory of retrograde menstruation suggests that endometrial cells flow backward through the fallopian tubes and into the peritoneal cavity during menses [2]. Other possible mechanisms include mesothelium, stem cells, mullerian rests, bone marrow stem cells, and embryonic vestiges as well as lymphatic or vascular dissemination and coelomic metaplasia [2].

Preoperative diagnosis for endometriosis affecting the appendix causing bowel obstruction is challenging, since the presenting symptoms and work-up with laboratory studies and imaging studies are generally non-specific. Adhesions, hernias, and neoplasms are typically placed high up on the differential for patients with a classical presentation of small bowel obstruction. Suspicion for an alternative diagnosis, such as endometriosis, especially in a reproductive-aged female should always be considered in patients with a virgin abdomen.

Since small bowel obstruction caused by endometriosis of the appendix is rare, it is often missed when evaluating a patient coming in with a small bowel obstruction. There are several cases of endometriosis of the appendix causing small bowel obstruction reported. In a systemic review of the literature, we found that similar to our case, in previously reported cases, timely and accurate preoperative diagnosis for the cause of small bowel obstruction was challenging given the vagueness of symptoms and work-up. Although the patients all presented with similar complaints (abdominal pain, nausea, emesis, and history of dysmenorrhea) and had similar outcomes, not all of them were managed conservatively initially. What made our case even more challenging and unique was the post-menopausal status of our patient. Extra-pelvic endometriosis is a rare clinical condition in post-menopausal women [5]. Given all these considerations, the etiology of the small bowel obstruction in our patient was difficult to determine.

Although preoperative diagnosis and determining the etiology of bowel obstruction are challenging, it is essential to do so in order to prevent further recurrence or complications. Endometriosis-related bowel obstruction should be suspected in all women, regardless of reproductive age or post-menopausal status, with vague abdominal complaints, a virgin abdomen, and a non-specific work-up who are coming in with obstructive symptoms and an unclear etiology for bowel obstruction based on their clinical history and imaging.

## Conclusions

Learning points

The majority of the cases of small bowel obstruction arise from mechanical obstruction, more specifically adhesions, hernias, and tumors. Once a patient is found to have small bowel obstruction, it is essential to determine the etiology of the obstruction for prompt treatment and to prevent further recurrence or complications. Endometriosis affecting the appendix is uncommon. Small bowel obstruction due to endometriosis affecting the appendix, although exceedingly rare, is possible. It is important to keep a broad differential in mind when working up a patient with a virgin abdomen for small bowel obstruction, as there are a vast number of etiologies of small bowel obstruction.
